# F-actin mechanics control spindle centring in the mouse zygote

**DOI:** 10.1038/ncomms10253

**Published:** 2016-01-04

**Authors:** Agathe Chaigne, Clément Campillo, Raphaël Voituriez, Nir S. Gov, Cécile Sykes, Marie-Hélène Verlhac, Marie-Emilie Terret

**Affiliations:** 1 CIRB, Collège de France, and CNRS-UMR7241 and INSERM-U1050, Equipe labellisée Ligue contre le Cancer, Paris F-75005, France; 2 Université Evry Val d'Essonne, LAMBE, Boulevard F Mitterrand, Evry 91025, France; 3 UMR7600-CNRS/UPMC, 4 Place Jussieu, Paris F-75005, France; 4 Department of Chemical Physics, Weizmann Institute of Science, Rehovot 76100, Israel; 5 CNRS-UMR168, Paris F-75248, France; 6 UPMC, 4 Place Jussieu, Paris F-75248, France; 7 Institut Curie, Centre de Recherche, Laboratoire Physico-Chimie, Paris F-75248, France

## Abstract

Mitotic spindle position relies on interactions between astral microtubules nucleated by centrosomes and a rigid cortex. Some cells, such as mouse oocytes, do not possess centrosomes and astral microtubules. These cells rely only on actin and on a soft cortex to position their spindle off-centre and undergo asymmetric divisions. While the first mouse embryonic division also occurs in the absence of centrosomes, it is symmetric and not much is known on how the spindle is positioned at the exact cell centre. Using interdisciplinary approaches, we demonstrate that zygotic spindle positioning follows a three-step process: (1) coarse centring of pronuclei relying on the dynamics of an F-actin/Myosin-Vb meshwork; (2) fine centring of the metaphase plate depending on a high cortical tension; (3) passive maintenance at the cell centre. Altogether, we show that F-actin-dependent mechanics operate the switch between asymmetric to symmetric division required at the oocyte to embryo transition.

## Abstract (web summary)

How the mitotic spindle is positioned in the centre of the cell during the first mitotic division is not clear. Here Chaigne *et al.* show that the pronucleus coarsely centres using F-actin/Myosin-Vb dynamics, and the metaphase plate is finely centred by an F-actin cage influenced by high cortical tension.

## Graphical abstract

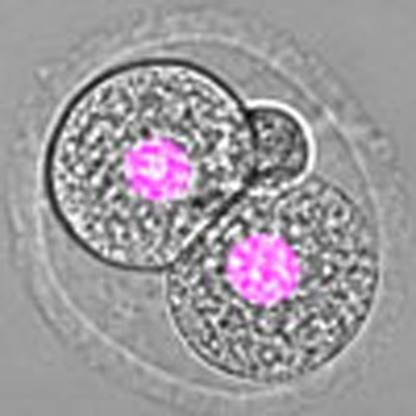

Mouse oocytes undergo a very asymmetric division in size during meiosis I. This asymmetry is a consequence of the migration of the microtubule spindle from the cell centre towards the closest cortex^1^. Oocytes are devoid of centrioles and astral microtubules^2^. As such, spindle positioning does not depend on microtubules^3^ as in most mitotic cells^4^, but on two actin networks. One is an F-actin cytoplasmic meshwork, nucleated by the cooperation between two types of actin nucleators, Spire1/2 and Formin-2 (refs 5, 6, 7, 8, 9, 10). It is present in Prophase I and dismantled at nuclear envelope breakdown (NEBD), maybe favouring meiotic spindle assembly in the absence of mechanical constraints^11^. This meshwork reforms progressively as meiosis I progresses. It is then composed of a dense and dynamic cytoplasmic F-actin meshwork and of an actin cage surrounding the microtubule spindle, connecting it to the cortex^5^,^9^,^12^,^13^. The other actin network shown to function during meiotic spindle positioning is a cortical F-actin thickening nucleated progressively by the Arp2/3 complex, which triggers the removal of Myosin-II from the cortex, promoting a drop of cortical tension^12^,^14^. The Arp2/3 complex lies downstream of a signalling cascade activated by Mos, a MAPKKK specific to the female gamete^15^,^16^. Myosin-II, localized at the poles of the actin cage^9^,^17^, pulls the spindle towards the cortex. However, since the spindle does not form at the exact cell centre^18^,^19^, one pole is closer to the cortex and thus pulled harder. The decrease in cortical tension allows the cortex to deform and to constitute a large overlap on which Myosin-II can further exert pulling forces, accelerating spindle migration^14^.

The first embryonic mitosis following fertilization in the mouse also occurs in the absence of true centrosomes and astral microtubules since the centrioles brought by the sperm are rapidly degraded^20^ and rebuilt *de novo* at the 64-cell stage^21^. This division is peculiar because the male and female DNAs are present in two different nuclei called pronuclei (we will refer to the period where both pronuclei are present as Pronucleus stage). They migrate from the periphery towards the centre of the embryo for several hours^22^,^23^ and are located roughly in the central region of the embryo at mitosis onset. They further undergo NEBD simultaneously, and then the two sets of chromosomes migrate and merge at the exact embryo centre, forming the metaphase plate. This division is symmetric, and requires F-actin since depolymerizing microfilaments before metaphase leads to an asymmetric division^24^,^25^. However, it is not known how F-actin is organized and controls the geometry of the division, how central positioning of the spindle is achieved and specifically whether spindle positioning also depends on the mechanical properties of the cortex as in oocytes. Yet, any deviation in spindle centring could perturb the symmetry of the division and compromise further embryo development and viability. We show here that F-actin-dependent mechanics are crucial for zygotic spindle positioning, ensuring first the coarse centring of pronuclei and second the fine centring of the metaphase plate, which is then maintained at the exact cell centre by a passive mechanism.

## Results

### Coarse centring of pronuclei depends on an F-actin meshwork

**Figure 1:**
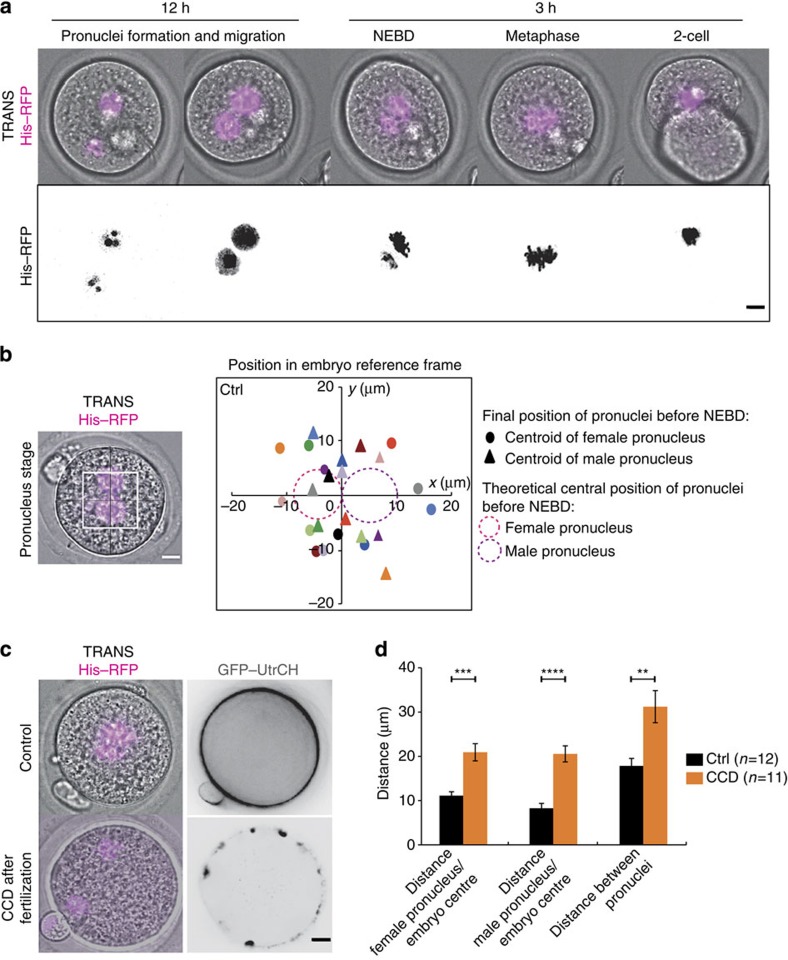
The coarse centring of the two pronuclei is F-actin dependent. (**a**) Control embryo expressing His–RFP (upper panel: purple, overlay on transmitted light, Z-projection over 20 μm; lower panel: black). The first two frames are 12 h apart then one picture is shown every 1 h (Scale bar: 10 μm). (**b**) Left panel: control embryo expressing His–RFP (purple, overlay on transmitted light, Z-projection over 20 μm) at the Pronucleus stage (scale bar, 10 μm). Right panel: dot plot showing the position of the centroid of the two pronuclei in the referential of the embryo (0 being the centre) 1 h before NEBD for 10 control embryos. The scale is represented by the white box on the left panel. Each colour represents an embryo, the circle is the female pronucleus and the triangle is the male pronucleus. The mean size of the maternal (pink) and paternal (purple) pronuclei is represented by the two dotted circles at the embryo centre. (**c**) Embryos just after NEBD expressing His–RFP (left panel, purple, overlay on transmitted light, Z-projection over 20 μm) and GFP–utrCH (right panel, black, one Z-plane is shown). Upper panel: control embryo; lower panel: embryo treated during pronuclei migration with 1 μg ml^−1^ Cytochalasin D (CCD). Scale bar, 10 μm. (**d**) Bar graph showing the male and female pronuclei distance to the embryo centre and to each other at NEBD for control embryos (black bars) or embryos treated during pronuclei migration with 1 μg ml^−1^ CCD (orange bars). Mean is shown of 12 controls and 11 CCD-treated embryos from 6 independent experiments. s.e.m. is plotted on each bar. Statistical significance of differences is assessed with *t*-test or *t*-test with Welch correction (*P* values: female pronuclei/embryo centre: 0.0004; male pronuclei/embryo centre <0.0001; between pronuclei: 0.0035).

To investigate the mechanism ensuring proper central position of the spindle, we performed *in vitro* fertilization and followed the first embryonic division in live confocal microscopy (Supplementary Movie 1, Fig. 1a). Following fertilization, the two pronuclei assemble at the periphery of the embryo (Supplementary Movie 1, Fig. 1a) and migrate towards the embryo centre in 12–15 h (Supplementary Movie 1, Fig. 1a). At the end of their migration, the two pronuclei are rarely at the perfect centre of the zygote, represented by the theoretical central position of the female and male pronuclei (Fig. 1b right panel, pink- and purple-dotted circles, respectively, as measured in ref. 26). They can be far apart as observed, for example, in the 30 μm distance separating the two orange spots (Fig. 1b right panel). Although it was hypothesized previously that the coarse centring could be F-actin dependent^22^, the precise mechanism for pronuclei centration has never been addressed. We first confirmed that the migration of the two pronuclei was indeed dependent on microfilaments by depolymerizing actin with 1 μg ml^−1^ Cytochalasin D after pronuclei formation but during their migration (Fig. 1c, right panel). In these embryos, NEBD occurred normally as seen on Fig. 1c (left panel) but pronuclei migration was abolished, leading to embryos with off-centred sets of chromosomes (Fig. 1c). Both male and female pronuclei end up mis-positioned with respect to the embryo centre and with respect to each other (Fig. 1d). Mis-positioning the pronuclei is sufficient to perturb the symmetry of the first zygotic division since when Cytochalasin D is washed out before NEBD there is a higher occurrence of asymmetric divisions (Supplementary Fig. 1a, quantification of the proportion of asymmetric divisions on Supplementary Fig. 1b).

**Figure 2:**
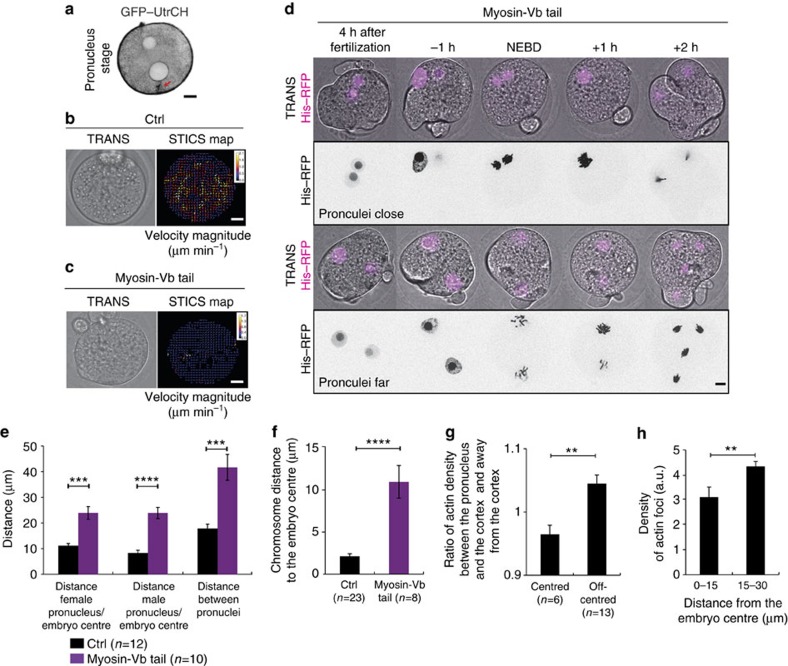
F-actin and Myosin-Vb-driven cytoplasmic flows position the pronuclei in the central region of the embryo. (**a**) Control embryo expressing GFP–UtrCH (black, one Z-plane is shown). The red arrow points at actin enrichment. Scale bar, 10 μm. (**b**,**c**) Left panels: embryos expressing (**c**) or not (**b**) Myosin-Vb tail. Right panels: associated vector maps of cytoplasmic flows obtained using STICS analysis. Heat bar unit in micrometres per minute. Scale bars, 10 μm. One example is shown out of 11 controls and 13 Myosin-Vb tail embryos. (**d**) Embryos expressing His–RFP (purple or black, Z-projection over 20 μm). Upper panels: pronuclei close; lower panels: pronuclei far. Scale bar, 10 μm. (**e**) Bar graph showing the pronuclei distance to the embryo centre and to each other 1 h before NEBD for controls (black bars) or Myosin-Vb tail-expressing embryos (purple bars). Mean is shown of 12 controls and 10 Myosin-Vb tail embryos. s.e.m. is plotted on each bar. Statistical significance of differences is assessed with a *t*-test or a *t*-test with Welch correction (*P* values: female pronuclei/embryo centre: 0.0005; male pronuclei/embryo centre <0.0001; between pronuclei: 0.0009). (**f**) Bar graph showing the chromosome mass distance to the embryo centre in metaphase for controls (black bar) and Myosin-Vb tail-expressing embryos (purple bar). Mean is shown for 23 controls and 8 Myosin-Vb tail embryos. s.e.m. is plotted on each bar. Statistical significance of differences is assessed with a Mann–Whitney test (*P* value <0.0001). (**g**) Graph bar showing the ratio of GFP–UtrCH fluorescent intensity between the pronucleus and the cortex and on the other side of the pronucleus. Mean is shown for 6 centred and 13 off-centred pronuclei embryos. s.e.m. is plotted on each bar. Statistical significance of the difference is calculated with a Mann–Whitney test (*P* value 0.0014). (**h**) Graph bar showing the mean percentage of actin-positive foci in each region of the embryo normalized by the effective surface of the region. Mean is shown from 9 embryos out of 2 independent experiments. s.e.m. is plotted on each bar. Statistical significance of the difference is calculated with a Mann–Whitney test (*P* values 0.007).

Since the migration of the two pronuclei is dependent on actin, we followed the dynamics of microfilaments in Pronucleus stage embryos (Supplementary Movie 2, Fig. 2a) using a green fluorescent protein–UtrCH (GFP–UtrCH) probe that specifically binds to F-actin^27^. At the Pronucleus stage, the embryo is filled with a very dense and dynamic F-actin mesh that seems to emanate from discrete foci, which could correspond to vesicles (Supplementary Movie 2) as described in oocytes^10^,^28^,^29^. These dots move at a mean speed of 10.55±3.70 μm min^−1^, a speed similar to the motion of actin-positive vesicles present in Prophase I oocytes (∼13 μm min^−1^)^28^,^29^. In Prophase I, the movement of the nucleus towards the cell centre is promoted by the active diffusion of actin-positive vesicles that establish a pressure gradient and is favoured by non-directed global cytoplasmic flows fluidizing the cytoplasm^29^. We thus checked for the presence of similar cytoplasmic flows in the zygote (Supplementary Movie 3) using spatiotemporal image correlation spectroscopy (STICs) analysis. As in Prophase I oocytes, we could detect the presence of non-organized cytoplasmic flows presenting a maximum speed of 2 μm min^−1^ (Fig. 2b). Consistent with the presence of cytoplasmic flows, inert fluorescent beads injected in early Pronucleus stage were found to move at a mean velocity of 18.2±7.8 μm min^−1^ from 2 to 3 h after pronuclei assembly (Supplementary Movie 4), close to the speed of the actin-positive foci.

In Prophase I, the active diffusion mechanism responsible for nucleus centring depends on the F-actin motor Myosin-Vb^29^. Indeed the movement of actin-positive vesicles induced by the activity of Myosin-Vb has a global effect, indirectly putting the cytoplasm in motion by dragging the fluid around moving vesicles^30^,^31^. To test if the same mechanism could be at play here, we expressed a Myosin-Vb tail which acts like a dominant negative construct for Myosin-Vb^10^. Myosin-Vb tail expressing embryos no longer display cytoplasmic flows (Supplementary Movie 5, Fig. 2c right panel), indicating that their cytoplasmic activity depends on Myosin-Vb activity. These embryos display massive cortical deformations (Supplementary Movies 6 and 7, Fig. 2d), but their pronuclei do not move from their cortical positions (Fig. 2d). Thus, when they undergo NEBD, they are mis-positioned with respect to the embryo centre and with respect to each other (Fig. 2d,e). Depending on the entry site of the spermatozoid, the two sets of chromosomes will start far or close from one another. When the two sets of chromosomes are close (Supplementary Movie 6, Fig. 2d, upper panel), they do gather after Myosin-Vb tail expression, but are improperly positioned since the metaphase plate ends up 10 μm away from the embryo centre, instead of 2 μm in controls (Fig. 2f). Strikingly, when the two sets of chromosomes are far away from each other (Supplementary Movie 7, Fig. 2d, lower panel), they are not able to merge and each pronucleus undergoes anaphase independently, leading to the formation of four haploid nuclei (Fig. 2d, lower panel). This phenotype recapitulates the Cytochalasin D phenotype (Fig. 1c,d) but with cortical deformations, which suggests that these cortical deformations are not responsible for the defects in pronuclei positioning observed after Myosin-Vb tail expression.

Altogether, these results argue that F-actin- and Myosin-Vb-driven cytoplasmic flows induce the motion of the two pronuclei during the Pronucleus stage, as it is the case in Prophase I oocytes that centre their nucleus. We then searched for what gives the directionality towards the centre to the movement.

### Mesh heterogeneities could drive coarse pronuclei centring

What could be the mechanism for coarse pronuclei centring? To address this question, we first searched for an anisotropy that could drive directional motion. Interestingly, the cytoplasmic actin meshwork does not seem homogeneous. Specifically, once the pronuclei have assembled under the cortex (Fig. 2a and referred in Fig. 2g as off-centred), the meshwork between the pronuclei and the adjacent cortex seems to be denser than the meshwork on the other side of the pronuclei (Fig. 2a red arrow). We then measured the fluorescence intensity of the F-actin probe (GFP–UtrCH) as a proxy for filament density between the pronuclei and the adjacent cortex (Fig. 2a red arrow) and on the other side of the pronuclei (see Methods). Before pronuclei migration, more actin is indeed present between pronuclei and cortex (Fig. 2g, the ratio is >1 for off-centred pronuclei). This is not the case once the pronuclei have migrated in the central region of the embryo, (Fig. 2g, the ratio is <1 for centred pronuclei). Close observation of the actin-positive foci revealed that there are no heterogeneities in their speed, but in their density, with more foci close to the cortex and less in the central region of the embryo (Fig. 2h). We can model the actin-positive foci as active particles propelled by Myosin-Vb, like for Prophase I oocytes^29^. Heterogeneities in actin density could be responsible for pronuclei centring. Indeed, similarly to a perfect gas, an assembly of *n* self-propelled particles induces a pressure that can be written: *P*=*nk*_B_*T*_e_, with *k*_B_ the Bolzmann constant and *T*_e_ the effective temperature of the particles. *k*_B_*T*_e_ is a measure of the agitation energy of the particles, and can be inferred from their mean squared speed (<*v*^*2*^>). The mean square speed of the actin-positive foci in embryos is uniform, however, their density is not (Fig. 2h). Such a gradient in the number of particles *n* would then result in a pressure gradient (∇*P*).

What would be the characteristics of such a pressure gradient? Here the resulting active force on a test object would be *F*_a_=∇*P.V*, with *V* the volume of the object to be moved. *V* is proportional to *R*^3^ with *R* the radius of the object. The force is counteracted by a friction force *F*_*f*_ proportional to *Rηv*_p_ (with *η* the viscosity and *v*_p_ the speed of the object). Hence, 
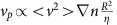
, which means that the speed of an object moving in the density gradient would be proportional to the square radius of this object: the bigger the object the higher the speed. This strong size dependence of the velocity predicted by the model may explain why pronuclei are effectively centred, while fluorescent beads are not (small fluorescent latex beads of 0.1 μm in diameter are not centred 10 to 12 h after pronuclei assembly, Supplementary Movie 8). Altogether, this suggests that a gradient of density of the actin-positive foci could be responsible for the coarse centring of big objects such as the two pronuclei.

### Chromosome merging determines the metaphase plate position

**Figure 3:**
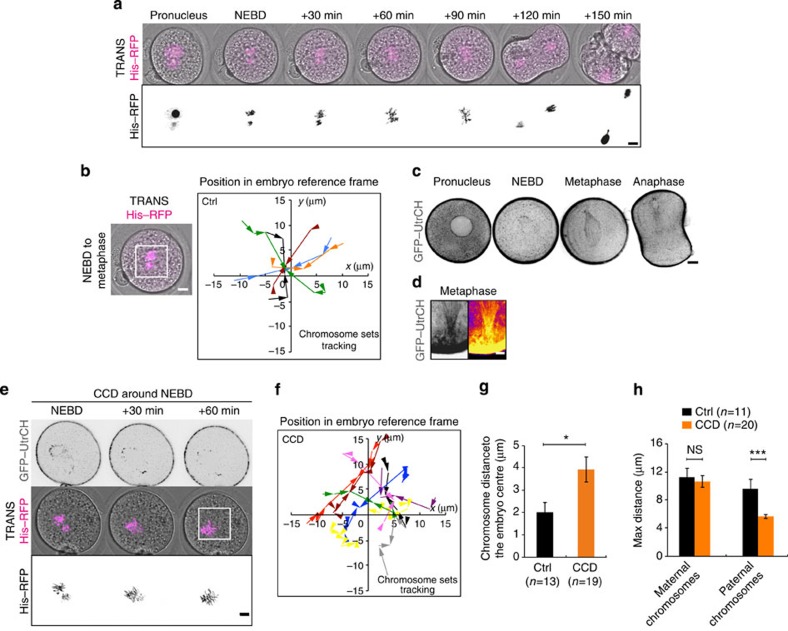
Fine chromosome centring in mitosis is actin dependent. (**a**) Control embryo expressing His–RFP (purple or black, Z-projection over 20 μm). Scale bar, 10 μm. (**b**) Left panel: Control embryo expressing His–RFP (purple, Z-projection over 20 μm) during chromosome migration. Scale bar, 10 μm. Right panel: graph showing the position of the centroid of the two sets of chromosomes in the referential of the embryo (0 being the centre) from NEBD to metaphase for controls. The scale is represented by the white box on the left panel. Each colour represents an embryo. The arrows point to the position where the chromosomes merge. One time point every 15 or 20 min over 1 h. (**c**) Embryo expressing GFP–UtrCH (black). One Z-plane is shown every 40 min. Scale bar, 10 μm. (**d**) Metaphase embryo expressing GFP–UtrCH (left: black. Right: white, higher intensity and black, lower intensity). Zoom on the connection between the actin cage and the cortex (cortex saturated to allow visualization of the actin cage filaments). Scale bar, 5 μm. (**e**) Embryo treated with 1 μg ml^−1^ Cytochalasin D (CCD) around NEBD expressing GFP–UtrCH (black, one Z-plane is shown) and His–RFP (purple or black, Z-projection over 20 μm). Scale bar, 10 μm. (**f**) Same as **b** for embryos treated with CCD around NEBD. (**g**) Bar graph showing the position of the centroid of the merged chromosomes for controls (black bar) and embryos treated with CCD around NEBD (orange bar). Mean is shown of 13 controls and 19 CCD embryos. s.e.m. is plotted on each bar. Statistical significance of differences is assessed with a Mann–Whitney test (*P* value 0.0175). (**h**) Graph showing the maximum distance (that is, the distance between the points furthest apart on the trajectories) of chromosome motion in controls (black bars, from Supplementary Fig. 1c) and embryos treated CCD around NEBD (orange bars) during the migration of the two sets of chromosomes towards the embryo centre. Mean of 11 controls and 20 CCD embryos are shown over 3 independent experiments. s.e.m. is plotted on each bar. Statistical significance of differences is assessed with *t*-test or Mann–Whitney tests (*P* values: female 0.6776; male 0.0004).

At mitosis onset, the two pronuclei are apposed more or less in the central region of the embryo (Fig. 1b). At NEBD, the two sets of chromosomes condense separately and are brought together in a slow but directed motion (roughly 1 h) (Supplementary Movie 9, Fig. 3a). The one-cell zygote is unique in this respect, having to achieve the fusion of maternal and paternal chromosome complements on a single metaphase plate^32^. Then the chromosome mass stays aligned in metaphase for roughly another hour before anaphase is triggered (Supplementary Movie 9, Fig. 3a). We followed precisely the position of the chromosomes during the first of these two phases, namely from NEBD to metaphase (Fig. 3b). During the migration of both sets of chromosomes (Fig. 3b), the two masses are brought almost towards the exact embryo centre (0 on the graph in Fig. 3b) with a mean maximum distance travelled of ∼10 μm both for the male and the female chromosomes (Supplementary Fig. 1c). At the end of this phase, the metaphase plate is positioned in close proximity with the exact embryo centre (Fig. 3b, almost all the curves converge around 0).

### Merging depends on microtubules but its position on F-actin

Since in mouse oocytes, spindle positioning depends on F-actin^5^,^6^,^7^,^8^,^9^,^10^,^12^, we investigated its role during mitosis in the zygote. We followed actin organization throughout the first mitosis (Supplementary Movie 10, Fig. 3c). At the Pronucleus stage, an F-actin meshwork fills the cytoplasm of the embryo (Figs 2a and 3c). The meshwork is dismantled around NEBD (Figs 1c and 3c, Supplementary Fig. 1d,e). Interestingly, such a dismantlement of F-actin meshwork has also been observed at meiosis resumption in oocytes^11^. The cytoplasmic meshwork then reforms and includes an F-actin cage, probably around the microtubule spindle (Fig. 3c), that is anchored at the cortex in metaphase (Fig. 3d). To test if F-actin plays any role in the merging of parental chromosomes, we acutely treated embryos just after NEBD with 1 μg ml^−1^ Cytochalasin D, sufficient to induce massive depolymerization of cytoplasmic F-actin (Fig. 3e, upper panel). Interestingly, F-actin depolymerization alters the location of the merging of chromosome (Supplementary Movie 11, Fig. 3e,f). Indeed, tracking of chromosomes indicates that their merging occurs on time (Fig. 3e) but rarely at the embryo centre (Fig. 3f). To assess the efficiency of the centring process, we measured the distance between the merged chromosomes and the embryo centre. In controls, the chromosomes merged around 2 μm away from the centre of the embryo (Fig. 3g, black bar). However, in embryos treated with CCD around NEBD, the chromosomes merge rather 4 μm away from the embryo centre (Fig. 3g), suggesting that F-actin is important for the proper positioning of the chromosome merge. Also, we measured the amplitude of chromosome displacement and observed that paternal chromosomes undergo a movement of smaller amplitude than controls (Fig. 3h). Altogether, these results show that F-actin participates in precise metaphase plate centring.

**Figure 4:**
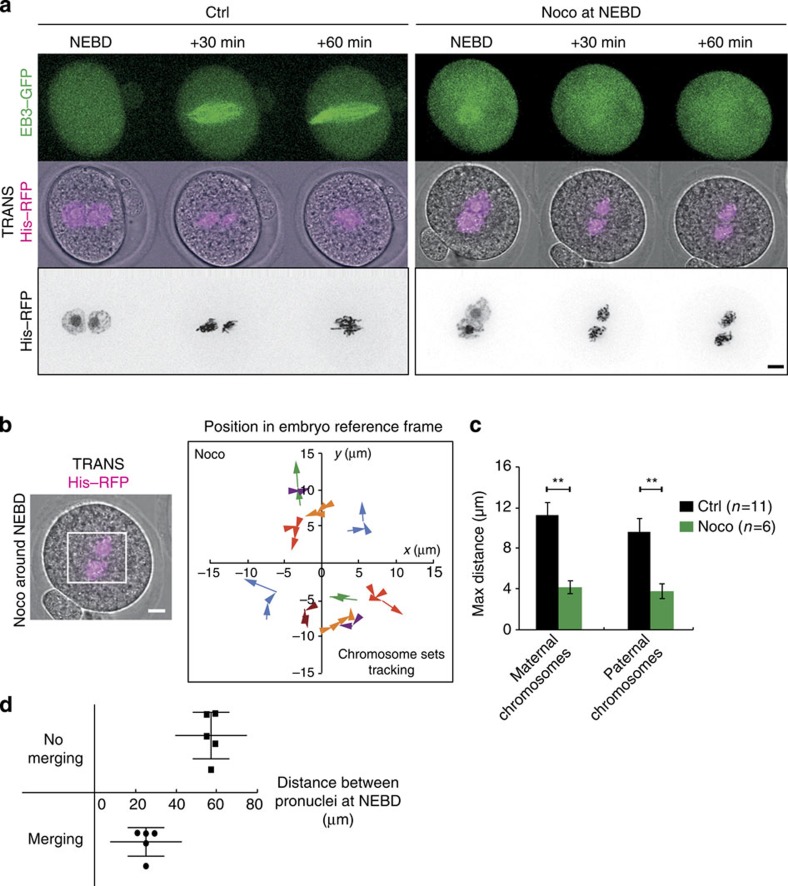
Chromosome merge is dependent on microtubules. (**a**) Control embryo (left panels) or embryo treated with 1 μM Nocodazole around NEBD (right panels) expressing EB3–GFP (upper panel, green, Z-projection over 20 μm) and His–RFP (purple or black, Z-projection over 20 μm). One image is shown every 30 min. Scale bar, 10 μm. (**b**) Left panel: Embryo treated with 1 μM Nocodazole around NEBD expressing His–RFP (purple, Z-projection over 20 μm) during the migration of the two sets of chromosomes. Scale bar, 10 μm. Right panel: graph showing the position of the centroid of the two sets of chromosomes in the referential of the embryo (0 being the centre) from NEBD for embryos treated with 1 μM Nocodazole around NEBD. The scale is represented by the white box on the left panel. Each colour represents an embryo. The arrows point to the last position recorded. Points are 30 min apart. (**c**) Graph showing the maximum distance (that is, the distance between the points furthest apart on the trajectories) of chromosome motion in controls (black bars, from Supplementary Fig. 1c) and embryos treated with 1 μM Nocodazole around NEBD (green bars) during the migration of the two sets of chromosomes towards the embryo centre (female: left bars; male: right bars). Mean of 11 controls and 6 Nocodazole-treated embryos are shown over 2 independent experiments. s.e.m. is plotted on each bar. Statistical significance of differences is assessed with Mann–Whitney tests (*P* values: female 0.0011; male 0.0011). (**d**) Dot plot showing if the two sets of chromosome merge as a function of initial chromosome distance at NEBD for 10 embryos expressing Myosin-Vb. Mean distance and s.e.m. are plotted for each condition.

Microtubules play a crucial role in spindle morphogenesis and positioning in most cells^33^, thus we decided to investigate their contribution to the process of chromosome merging. We therefore treated embryos after pronuclei migration, around NEBD, with 1 μM Nocodazole and monitored the position of chromosome complements (Supplementary Movie 12, Fig. 4a). The Nocodazole treatment induced extensive microtubule spindle depolymerization after 30 min (Fig. 4a). Strikingly, parental chromosomes never merge onto a unique metaphase plate in the absence of microtubules (Supplementary Movie 12, Fig. 4a,b). Precise tracking of the two sets of chromosomes indicates that they undergo a non-oriented motion (Fig. 4b), with a maximum distance travelled smaller than in controls (Fig. 4c). Altogether, these results suggest that maternal and paternal chromosomes are brought together in a microtubule-dependent manner. Since Myosin-Vb tail expressing embryos harbour pronuclei that can be close or far apart (Fig. 2d), following chromosome merging in these embryos allows to gain insight into the reach of microtubules. Indeed, when the two sets of chromosomes are separated by <40 μm they are able to merge, whereas when they are >40 μm apart their merging fails (Fig. 2d, quantification on Fig. 4d). Therefore the microtubules are not capable of bridging a longer distance than 40 μm, possibly explaining why the pronuclei have first to be coarsely centred via an F-actin-dependent mechanism in order to merge.

### Myosin-II drives cortical tension increase at NEBD

**Figure 5:**
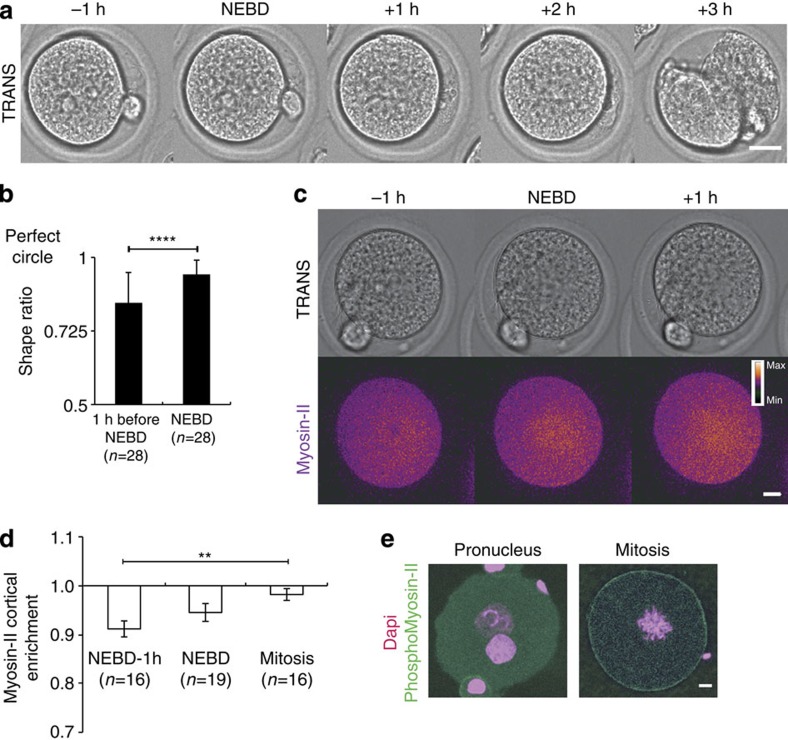
Embryos round up at NEBD following a Myosin-II-dependent cortical tension increase. (**a**) Embryo before and during the first mitosis. One Z-plane is shown every 1 h. Scale bar, 10 μm. (**b**) Graph bar showing the shape ratio (see Methods) of embryos before and during mitosis. Mean is shown of 28 embryos from 10 independent experiments. s.d. is plotted on each bar. Statistical significance of differences is assessed with a Mann–Whitney test (*P* value <0.0001). (**c**) Embryo observed by transmitted light (upper panel) expressing SF9–GFP (Myosin-II intrabody, blue: lower intensity, orange: higher intensity). One Z-plane is shown every 1 h. Scale bar, 10 μm. (**d**) Bar graph showing the ratio between the average intensities of cortical versus cytoplasmic Myosin-II in embryos expressing SF9–GFP 1 h before NEBD, at NEBD or during mitosis. For each embryo, six measurements were taken in the cortex and six in the cytoplasm (see Methods). Mean of 16 embryos 1 h before NEBD, 19 at NEBD and 16 mitotic embryos is shown assessed over 6 independent experiments. S.e.m. is plotted on each bar. Statistical significance of differences is assessed with a *t*-test (*P* values: 1 h before NEBD/NEBD 0.1854; NEBD/mitotic 0.1066; 1 h before NEBD/mitotic 0.0013). (**e**) Control embryo at the Pronucleus stage (left panel) or during mitosis (right panel) stained for chromosomes (magenta, Z-projection is shown over 25 μm) and PhosphoMyosin-II (green, one Z-plane is shown). Scale bar, 5 μm.

Cortical tension being essential for spindle positioning in mouse oocytes^12^,^14^, we decided to investigate whether it also regulates spindle centring in embryos. Like other cell types, mouse zygotes round up at mitosis onset (Supplementary Movie 13 and Fig. 5a,b). Opposite to the measurements made in oocytes where cortical tension decreases during the division^12^,^34^, cortical tension does increase during mitosis (Table 1) as in other mitotic cells^35^. Interestingly, even if the increase in cortical tension is modest (2-fold, Table 1) compared with the important (30-fold) drop experienced by oocytes during meiosis I (Table 1)^12^ it is sufficient to induce the rounding up of extremely deformed embryos. This is the case, for example, in embryos expressing the Myosin-Vb tail (Fig. 2d and Supplementary Movies 6 and 7). To decipher the molecular basis for this change in cortex tension, we followed the localization of Myosin-II^36^, a major regulator of tension in cells^37^,^38^,^39^, in particular in oocytes^12^,^14^,^34^. Myosin-II is present in the cytoplasm both at the Pronucleus stage and throughout the first mitotic division (Supplementary Movie 14, Fig. 5c). However, during mitosis it is slightly enriched at the cell cortex (Supplementary Movie 14, Fig. 5c,d, a ratio closer to 1 corresponds to cortex enrichment). The cortical recruitment of PhosphoMyosin-II at mitosis onset was also observed on fixed embryos (Fig. 5e). The cortical recruitment of Myosin-II is modest compared with its enrichment in Prophase I oocytes^12^. This is consistent with the amplitude of cortical tension variations observed during meiosis I in oocytes versus the one observed here in the zygote (Table 1). Altogether, our results suggest that differently from oocytes^12^,^14^ and similarly to other cell types^39^, Myosin-II is recruited at the cortex at mitosis onset in one-cell embryos, leading to an increase in cortical tension.

### Central merging relies on a high cortical tension

**Figure 6:**
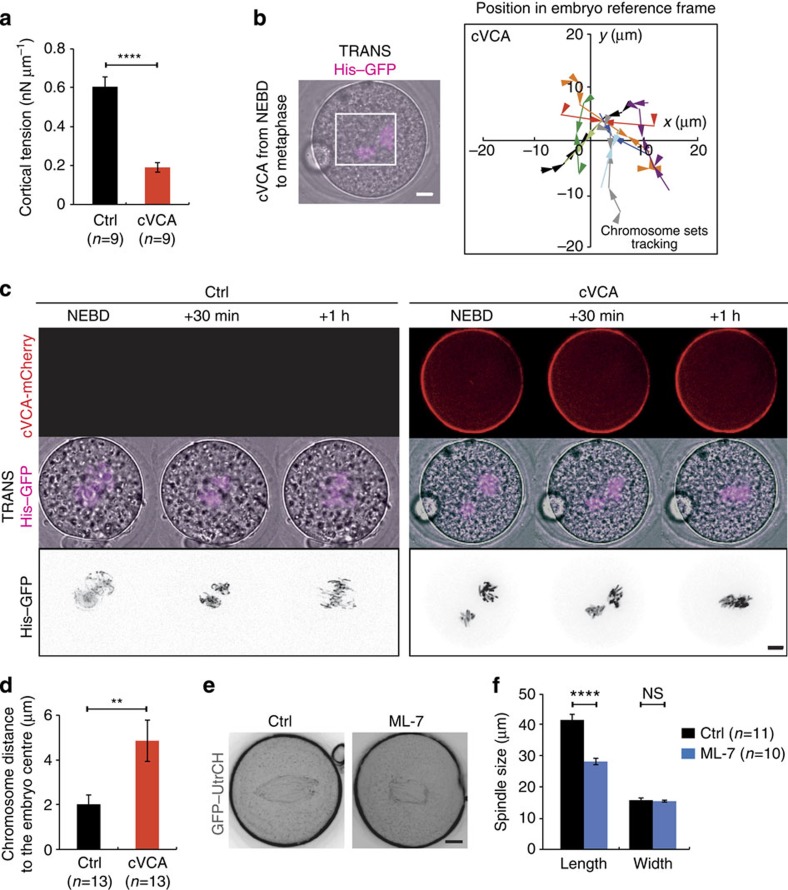
Decreasing cortical tension impairs the fine centring of the chromosomes. (**a**) Bar graph showing the values of cortical tension of a mitotic embryo expressing cVCA (red bar) or not (black bar). Mean is shown over two independent experiments. s.e.m. is plotted on each bar. Statistical significance of differences is assessed with a *t*-test (*P* value <0.0001). (**b**) Left panel: Embryo expressing His–RFP (purple, Z-projection over 20 μm) together with cVCA during the migration of the two sets of chromosomes. Scale bar, 10 μm. Right panel: graph showing the position of the centroid of the two sets of chromosomes in the referential of the embryo (0 being the centre) from NEBD to metaphase for representative embryos expressing cVCA. The scale is represented by the white box on the left panel. Each colour represents an embryo. The arrows point to the position where the two sets of chromosomes merge. One time point is shown every 30 min. (**c**) Embryos expressing His–RFP alone (left panels) or together with cVCA (right panels). Upper panel: cVCA (red), one Z-plane is shown; middle and lower panels: His–RFP, purple or black, Z-projection over 20 μm. One frame is shown every 30 min. Scale bar, 10 μm. (**d**) Bar graph showing the position of the centroid of the merged chromosomes for controls (black bar) and embryos expressing cVCA (red bar). Mean is shown of 13 controls and 13 embryos expressing cVCA. s.e.m. is plotted on each bar. Statistical significance of differences is assessed with a Mann–Whitney test (*P* value 0.0015). (**e**) Embryos expressing GFP–UtrCH (black) treated (right panel) or not (left panel) in metaphase with 30 μM ML-7. Scale bar, 10 μm. (**f**) Bar graph showing the mean length and width of the actin cage in metaphase for 11 controls (black bars) and 10 ML-7 (blue bars) treated embryos from two independent experiments. s.e.m. is plotted on each bar. Statistical significance of differences is assessed with a *t*-test (*P* value length <0.0001) or a Mann–Whitney test (*P* value width 0.3167).

To investigate the role of the increase in cortical tension, we decided to artificially decrease it using a membrane-targeted cVCA, a construct promoting F-actin nucleation exclusively at the cortex and triggering cortical Myosin-II removal, thus lowering cortical tension, as observed in oocytes^14^. The cVCA efficiently decreased cortical tension during zygote's mitosis (Fig. 6a). The amplitude of the decrease in cortical tension due to cVCA expression was, however, smaller compared with the one measured during meiosis (3-fold decrease compared with 100-fold^14^). Interestingly, the mechanism responsible for the drop in cortical tension was comparable to the one present in oocytes. First, cVCA-expressing embryos harboured a cortical F-actin thickening (Supplementary Fig. 2a) of 2.5 μm compared with a thickness of 1 μm in controls (Supplementary Fig. 2b). The size and width of the actin cage were unchanged arguing that here, as for oocytes, the membrane-targeted cVCA construct acted mostly at the cortex and not indirectly on the cytoplasmic actin mesh (Supplementary Fig. 2c). Second, cVCA expression was sufficient to impair Myosin-II recruitment at the cortex (Supplementary Fig. 2d,e: the level of Myosin-II at the cortex is constant in embryos expressing cVCA between Pronucleus stage and mitosis, whereas it increases in mitosis in control embryos). cVCA-expressing embryos harbour properly positioned pronuclei (Supplementary Fig. 2f), reinforcing the idea that pronuclei centring could depend on F-actin-driven active diffusion, this process being also tension independent in oocytes^29^. Importantly, none of the defects are due to the presence of Ezrin in the cVCA construct, since embryos solely overexpressing Ezrin behave like controls (Supplementary Figs 2f and 4h).

In cVCA-expressing embryos, the two chromosome complements merge with timings comparable to controls (Supplementary Movie 15, Fig. 6c, quantification on Supplementary Fig. 2g). However, the position of the merge is not at the exact cell centre (Fig. 6b–d). Indeed, precise quantification of the trajectories shows that parental chromosomes merge up to 10 μm away from the embryo centre (Fig. 6b, purple curve, for example; almost no curves converge at 0). This was very different to what was observed in controls (Fig. 3b). Interestingly, the lack of precise centring is not due to a reduction in chromosome motion since the mean maximal distance travelled by chromosomes is comparable to controls (Supplementary Fig. 2h). These results demonstrate that a high cortical tension defines the exact embryo centre where maternal and paternal chromosomes will eventually merge.

Both decreasing cortical tension as well as depolymerizing F-actin lead to merging of the chromosomes away from the perfect embryo centre. This suggests that cortical tension is transmitted to the chromosomes via microfilaments. The F-actin cage surrounds the chromosomes and is connected to the cortex, it could thus transmit forces to the spindle. What is the molecular mechanism ensuring this relay of tension from the cortex to the spindle? To address this question, we observed Myosin-II localization, which is responsible for F-actin-based force generation, in particular in mouse oocytes^9^,^12^,^14^. A closer inspection of the active form of Myosin-II staining in mitosis, PhosphoMyosin-II, showed that it is present at spindle poles, colocalizing with pericentrin (Supplementary Fig. 3a, yellow foci at the poles of the spindle) as in meiosis I where it is responsible for spindle traction to the cortex^9^,^12^,^17^. We thus wondered whether Myosin-II could put this F-actin cage under tension. To address this question, we treated one-cell zygotes in metaphase with ML-7, which reduces Myosin-II activity^40^. Less than 20% of embryos treated with 30 μM ML-7 undergo normal cytokinesis (Supplementary Fig. 3b), consistent with a role for Myosin-II in cytokinesis^41^. Embryos treated with ML-7 have shorter actin cages (Fig. 6e,f), suggesting that Myosin-II puts the actin cage under tension. ML-7 does not act on the actin cage by modulating the amount of microfilaments connected to the cage, since the density of F-actin (relative to the size of the cage), quantified using the fluorescence level of GFP–UtrCH, is comparable in controls versus ML-7-treated embryos (Supplementary Fig. 3c). Therefore, Myosin-II may put the actin cage under tension in metaphase. Altogether, these results suggest that Myosin-II could transmit the forces exerted by the increase in cortical tension to the microtubule spindle via the F-actin cage, placing it at the exact embryo centre.

### Maintenance of the chromosomes at the cell centre is passive

**Figure 7:**
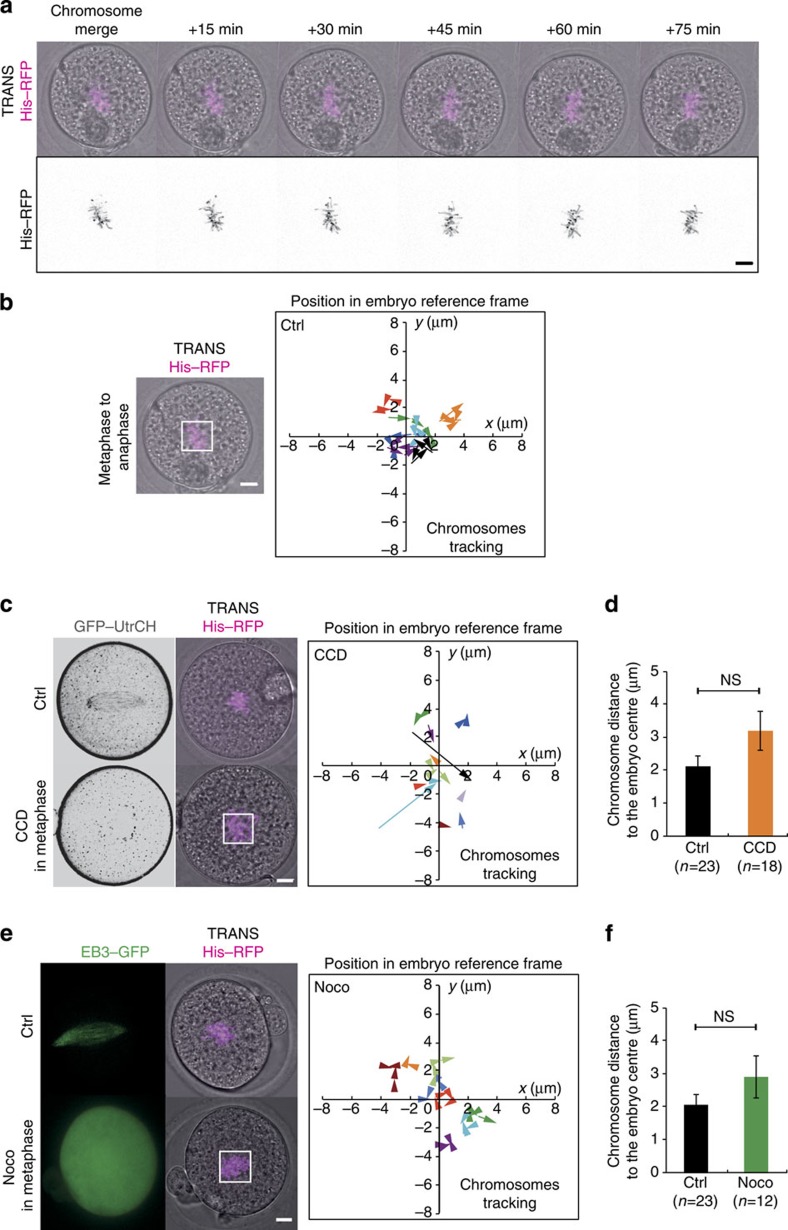
Maintenance of the central position of the metaphase plate is actin and microtubule independent. (**a**) Control embryo expressing His–RFP (purple or black, Z-projection over 20 μm). One picture is shown every 15 min. Scale bar, 10 μm). (**b**) Left panel: Control embryo expressing His–RFP (purple, Z-projection over 20 μm) after chromosome merging. Scale bar, 10 μm. Right panel: graph showing the position of the centroid of the chromosomes in the referential of the embryo (0 being the centre) from metaphase to anaphase for representative control embryos. The scale is represented by the white box on the left panel. Each colour represents an embryo. The arrows point to the last position recorded before anaphase. One time point is shown every 15 or 20 min. (**c**) Left panel: control metaphase embryo (upper panel) and embryo treated with 1 μg ml^−1^ Cytochalasin D (CCD) in metaphase (lower panel) expressing GFP–UtrCH (black, one Z-plane is shown) and His–RFP (purple, Z-projection over 20 μm). Scale bar, 10 μm. Right panel: Same as **b** for embryos treated with CCD in metaphase. (**d**) Bar graph showing the chromosome mass distance to the embryo centre in metaphase for controls (black bar) and embryos treated with CCD in metaphase (orange bar). Mean is shown of 23 controls and 18 CCD-treated embryos from 2 independent experiments. s.e.m. is plotted on each bar. Statistical significance of differences is assessed with a Mann–Whitney test (*P* value 0.204). (**e**) Left panel: control metaphase embryo (upper panel, same as in Fig. 4a) and embryo treated with 1 μM Nocodazole in metaphase (lower panel) expressing EB3–GFP (green, one Z-plane is shown) and His–RFP (purple, Z-projection over 20 μm). Scale bar, 10 μm. Right panel: Same as **b** for embryos treated with 1 μM Nocodazole in metaphase. (**f**) Bar graph showing the chromosome mass distance to the embryo centre in metaphase for controls (black bar) and embryos treated with Nocodazole in metaphase (green bar). Mean is shown of 23 controls and 12 Nocodazole-treated embryos from 2 independent experiments. s.e.m. is plotted on each bar. Statistical significance of differences is assessed with a Mann–Whitney test (*P* value 0.2203).

Once the chromosomes have merged and are positioned at the exact embryo centre, they stay at this position until anaphase (Figs 1a and 3a). We precisely monitored and tracked the position of the chromosomes during metaphase in control embryos (Fig. 7a,b) and showed that they undergo very little motion until anaphase (Fig. 7a,b). Thus, the position where the chromosomes merge specifies the division plane. To test whether F-actin is involved in maintaining the metaphase plate at the embryo centre, we acutely depolymerized cytoplasmic F-actin in metaphase with 1 μg ml^−1^ Cytochalasin D (Fig. 7c, lower panel). This treatment has little incidence on the motion of chromosomes (Supplementary Movie 16; Fig. 7c, right panel; Supplementary Fig. 4a), despite more variability in the amplitude of chromosomes movements in CCD-treated embryos (Supplementary Fig. 4a). However, the position of chromosomes before anaphase is comparable to controls (Fig. 7d). Thus the overall maintenance of the spindle at the cell centre is actin independent. Consistent with this result, when Myosin-II is inhibited, the chromosomes stay centrally located (Supplementary Fig. 4b), indicating that the tension imposed by Myosin-II to the cage is not necessary for the maintenance of the spindle at the centre of the cell.

We then tested the contribution of microtubules in this process by adding 1 μM Nocodazole in metaphase. Similarly, we could not detect any effect on the position of the metaphase plate (Supplementary Movie 17, Fig. 7e,f, Supplementary Fig. 4c). These results show that the global spindle maintenance at the embryo centre is microtubule independent. Eventually, when both microfilaments and microtubules were depolymerized in metaphase, the central position of the chromosomes was unaffected (Supplementary Movie 18, Supplementary Fig. 4d,e). In this condition, however, the chromosomes moved slightly less than in controls (Supplementary Fig. 4f), probably due to the fact that the microtubule spindle promotes slight chromosomes oscillations as in mitotic cells^42^.

**Figure 8:**
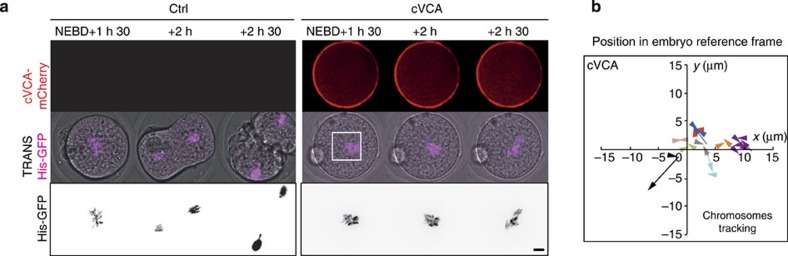
Maintenance of the central position of the metaphase plate does not rely on tension but could depend on a viscous cytoplasm. (**a**) Embryos expressing His–RFP alone (left panels) or together with cVCA (right panels). Upper panel: cVCA (red), one Z-plane is shown; middle and lower panels: His–RFP, purple or black, Z-projection over 20 μm. One frame is shown every 30 min. Scale bar, 10 μm. (**b**) Graph showing the position of the centroid of the chromosomes in the referential of the embryo (0 being the centre) from metaphase to anaphase for representative embryos expressing cVCA. The scale is represented by the white box in Fig. 8a. Each colour represents an embryo. The arrows point to the last position recorded before anaphase. One time point is shown every 20 or 30 min.

We also followed metaphasic chromosomes in embryos with artificial reduction in cortical tension. As shown above, their chromosomes do merge, but not at the embryo centre (Supplementary Movie 15, Figs 6b,c, 8a). Following their gathering, during metaphase the chromosomes undergo small movements as in controls (Supplementary Movie 15, Fig. 8b), with a maximal amplitude similar to controls (Supplementary Fig. 4g). Thus in metaphase, the chromosomes stay at the position where they merged, that is, slightly off-centre (Supplementary Movie 15, Fig. 8b). Altogether, these results suggest that a high cortical tension is essential for accurate merging of maternal and paternal chromosomes at the exact embryo centre, but is not necessary for spindle maintenance there.

Since spindle maintenance at the cell centre does not seem to be achieved via active cytoskeletal mechanics, we wondered whether viscosity could be impeding on spindle motion. We thus measured viscosity using a micropipette aspiration technique (see Methods) and observed that it increases in metaphase up to threefold its value at the Pronucleus stage (Table 1). Accordingly, when beads were injected in embryos they were found to move fast at the early and late Pronucleus stages (Supplementary Movies 4–8; mean bead velocity of 18.5±7.8 μm min^−1^ for early and 17.2±9.4 μm min^−1^ for late Pronucleus stage) but significantly slower during mitosis (Supplementary Movie 19; mean bead velocity of 13.8±9.9 μm min^−1^; Mann–Whitney test *P* values: early versus mitosis <0.0001; late versus mitosis 0.0013). Therefore maintenance of spindle position during metaphase could be due to a passive mechanism, a consequence of an increased viscosity in mitosis.

## Discussion

Mouse zygotes are not polarized, and the positioning of the first cleavage plane depends only on the position of the two pronuclei^43^. However, how the two pronuclei are positioned has never been precisely addressed. Some studies suggested that it was actin dependent^22^ whereas studies in other model systems pointed to a microtubule-dependent mechanism^44^. Here we unambiguously show that coarse pronuclei centring in mouse embryos depends on a dynamic F-actin/Myosin-Vb meshwork but not on cortical tension, suggesting that pronuclei centring could occur via an actin-based mechanism similar to the one described in Prophase I mouse oocytes (Supplementary Fig. 5)^29^. Interestingly, in *Arabidopsis thaliana*, also lacking centrosomes, the male nucleus migrates toward the female nucleus in an actin-myosin dependent manner^45^. This suggests that mechanisms of nucleus positioning that rely only on F-actin could be common to all cells that are devoid of centrioles.

Importantly, previous works using *in vitro* systems established that in metaphase, due to the dynamic instability of microtubules, spindle size scales with cell size, suggesting an upper limit for spindle reach around 40 μm in cells of a size comparable to oocytes^46^,^47^,^48^, which is exactly the maximal distance for proper chromosome merging *in vivo* here. Altogether, this strongly argues that the F-actin-dependent coarse centring of pronuclei is absolutely required to bring the two chromosome complements within range of microtubule reach. Indeed, even in the huge starfish oocyte that does possess centrioles, the microtubules are too short to capture the chromosomes and an F-actin fishnet is responsible for their gathering^49^,^50^.

However, this is a rather coarse centring and how the perfect symmetry is achieved has never been investigated. We propose that the main event controlling acute centring of the spindle is a centrally oriented gathering of the two sets of chromosomes, which requires F-actin and a Myosin-II-dependent increase in cortical tension (Supplementary Fig. 5). The gathering itself relies on microtubules, but the position of the gathering depends on an increase of cortical tension and the presence of F-actin. The formation of an F-actin cage connected to the cortex (Fig. 3c,d), probably put under tension by Myosin-II at its poles (Supplementary Fig. 3a, Fig. 6e,f) and thus relaying cortical tension, could therefore allow the chromosomes to be centrally positioned when they merge, acting like a spring. The precise mechanism remains to be determined. Interestingly, chromosome mis-positioning is never drastic (Figs 3f and 6b), which likely results from the inherent geometry of the system: a long microtubule spindle (Fig 4a) and a long F-actin cage (Figs 3c and 6e) anchored to the cortex that cannot move from more than a few microns.

We also hypothesize here how, after chromosome merge, they are maintained at their central position. Neither F-actin nor microtubules or cortical tension play any role in this process. We show that a passive mechanism relying on an increase in cytoplasmic viscosity could be at play. Interestingly, this is very different from meiosis I where the spindle is dragged from the centre towards the cortex of the oocyte in an ∼10 times less viscous cytoplasm^14^. Thus the viscosity of the cytoplasm could be differently modulated in these two systems to allow opposite functions regarding spindle positioning. In the future, it will be interesting to determine how actin meshes organized during M-phase can end up increasing or reducing cytoplasmic viscosity to influence organelle positioning.

Strikingly, similar cellular processes involving F-actin and cortical tension are used to position the spindle at the cell periphery in oocytes and at the cell centre in one-cell embryos. One difference is the presence or not of the upstream regulator, Mos, which is absent in embryos^15^,^16^. Thus, it could imply that Mos, a specificity of the female gamete from jelly fish to mammals^1^,^51^, degraded after fertilization^52^,^53^,^54^, could participate in the switch from an oocyte to an embryo mode of division.

## Methods

### *In vitro* fertilization microinjection and live imaging

*In vitro* fertilization was performed by releasing the sperm in home-made Fraser medium^55^ and letting it capacitate for 1.5–2 h, then releasing 5–10 μl of capacitated sperm on Metaphase II oocytes from 5–8-week-old OF1 females stimulated around 19:00 with 50 IU of pregnant mare serum and 50 IU human chorionic gonadotropin at 48-h interval. Fertilization was allowed for 2–4 h typically from 14:00 to 16:00 and then embryos were transferred and cultured in M2 medium. Embryos were micro-injected at the Pronucleus stage with complementary RNAs (cRNAs) using an Eppendorf Femtojet micro-injector^56^. Culture and imaging were carried out under oil at 37 °C. Due to asynchrony of fertilization, embryos initiated mitosis during a 4-h period. The chromosome state was observed to infer mitotic progression. Two different females minimum were used for each experiment, and experiments were replicated at least twice to confirm the reproducibility of the results. The animal facility scientific council of the CIRB granted permission for the animal experiments described here.

Measurements of cortical tension in embryos were performed as previously described for oocytes^12^. Briefly, the zona pellucida of embryos was removed using acid tyrod (pH=2.3), and embryos were analysed at desired stages. Embryos were loaded onto a chamber equilibrated with M2+BSA medium. A glass micropipette of a diameter five times smaller than the embryo diameter was connected to a water reservoir of adjustable height to apply a defined aspiration pressure. Zero aspiration pressure was set prior to each experiment by checking the absence of visible flow inside the pipette. Observations were made through an inverted microscope (Axiovert 200, Zeiss) equipped with a × 40 immersion oil objective (Neofluar 1.3 numerical aperture (NA)) and connected to a charge-coupled device (CCD) camera (XC-ST70CE, Sony, Japan). For every applied pressure, we monitored the length *L* of the embryo portion aspirated in the pipette as a function of time and derived the speed d*l*/d*t* at which the embryo cortex enters the pipette. Then, we extrapolated the critical aspiration pressure Δ*P*_c_ at which d*l*/d*t*=0 from the plot d*l*/d*t* versus the aspiration pressure. To obtain the cortical tension *T*_c_, we used the ‘viscous drop' model previously used for cells^12^ that gives,


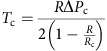


where *R* is the pipette radius and *R*_c_ is the cell radius.

Spinning disk images were acquired using a Plan-APO × 40/1.25 NA objective on a Leica DMI6000B microscope enclosed in a thermostatic chamber (Life Imaging Service) equipped with a CoolSnap HQ2/CCD camera (Princeton Instruments) coupled to a Sutter filter wheel (Roper Scientific) and a Yokogawa CSU-X1-M1spinning disc^57^. The Metamorph software (Universal Imaging) was used to collect data and ImageJ (NIH) to analyse and process data. Since the embryo is a round cell with two objects of interest, the two sets of parental DNA, all analysis were performed in two dimensions in the medial plane formed by these two objects.

### Plasmids and *in vitro* transcription of cRNAs

We used the following constructs: pspe3–GFP–UtrCH^5^, pRN3-histone–red fluorescent protein (pRN3-histone–RFP)^58^, pRN3-SF9–GFP^36^, pRN3-EB3–GFP^59^, pRN3-EzTD-mCherry-VCA^14^, pRN3-histone–GFP^14^ and pGEMHE-mCherry-Myosin-Vb (gift from Melina Schuh, MRC Cambridge, UK)^10^,^29^.

*In vitro* synthesis of capped cRNAs was performed using the mMessage mMachine kit (Ambion) and the RNeasy kit (Qiagen) following the manufacturer's instructions^60^. cRNAs were centrifuged at 4 °C for 45 min at 20,000*g* before microinjection.

### Trajectories, STICS analysis and trackings

Embryos were followed from NEBD until anaphase in two dimensions and only the ones with chromosomes moving in the focal plane and that divided in the focal plane were quantified. The position of the centroid of the two sets of chromosomes (male and female) was tracked using Metamorph until their merging, then the position of the centroid of the chromosome mass was recorded. The position of the centroid of the embryo was recorded at each time point. The trajectories were drawn by calculation of the coordinates of the centroid of chromosomes in the embryo frame of reference. The tracking of actin-positive foci and STICS analysis were performed as previously described^29^. Briefly, GFP–UtrCH-expressing embryos were denoised using the Safir plugin for Metamorph, background was subtracted, the images were realigned using the StackReg plugin of Fiji for embryos expressing GFP–UtrCH and Linear Stack Alignment with SIFT for transmitted images, and corrected for bleaching if required on Fiji. Tracking was performed using the TrackMate plugin of Fiji. Heat maps were generated using the ImageJ plugin ‘ICS tools: STICS map jru v2' using regions avoiding the cortex. Controls are taken from different experiments (the black bars on Figs 3h and 4c, and Supplementary Fig. 2h come from Supplementary Fig. 1c; the controls on Figs 3g and 6d are the same; the controls on Fig. 7d,f and Supplementary Fig. 4b are the same; the controls on Supplementary Fig. 4a,c,f,g are the same).

### Actin vesicles density analysis

To assess the difference in actin intensity between cortex and cytoplasm close to the pronuclei, GFP–UtrCH fluorescence intensity was measured in embryos in a defined round region. For each pronucleus, four measures were taken between the cortex and the pronucleus, and four between the pronucleus and the adjacent cytoplasm, all around the pronucleus. The ratio between the sum of fluorescence intensities on the cortex side and the sum of fluorescence intensities on the cytoplasm side is then calculated.

To assess the density of actin-positive foci on each region, the number of foci in each region was extracted using the Trackmate analysis described above. The percentage of foci in each region over the total number of foci in each embryo was calculated. The effective surface of the region was calculated by measuring the surface occupied by each pronucleus. The actin-positive foci density is the ratio of these two values.

### Drug treatments

Embryos were treated with 1 μM Nocodazole^1^,^5^ (Sigma, Ref. M1404), 1 μg ml^−1^ Cytochalasin D (Life Technologies, Ref PHZ1063)^1^,^5^ or 30 μM ML-7 (refs 9, 12) (Sigma, Ref. I 2764), diluted in M2 medium. Treatments were performed during pronuclei migration, around NEBD or in early metaphase. The controls presented in all panels represent control embryos extracted from different experiments.

Latex fluorescent beads (0.1 μm; Life Technologies, F8803) were rinsed several times in nuclease-free water before use to remove traces of sodium azide and diluted 500 times before injection to avoid aggregates.

### Myosin-II and F-actin measurements

The overall thickness of both the cortical outer bright layer and the cortical inner dimmer layer (which increases in cVCA-expressing embryos) was measured manually in Metamorph. All measures were taken on one focal plane corresponding to the embryo's biggest diameter to avoid projection artifacts as done in ref. 9.

To assess the cortical enrichment of Myosin-II, SF9–GFP fluorescence intensity was measured in embryos, after background subtraction, in a defined square region smaller than cortex width. For each embryo, six measures were taken in the cortex and six in the cytoplasm, all around the embryo. The cortical enrichment is then defined as the ratio between the sum of fluorescence intensities in the cortex and the sum of fluorescence intensities in the cytoplasm as done in ref. 9.

### Volume measurements

Pictures were taken 2 μm apart along 100 μm to cover all embryo volume on GFP–UtrCH-expressing embryos as a marker for cortex borders. The volume of the two daughter cells was calculated using the Measure stack plugin for ImageJ (Copyright (c) 2002, 2005, OptiNav, Inc.).

### Shape ratio measurements

For each embryo, the inner and outer radius were measured. The shape ratio is defined as the ratio between these inner and outer radius, such as the closer the ratio to 1, the rounder the cell.

### Immunofluorescence

The zona pellucida of Pronucleus stage or mitotic embryos was removed using Acid Tyrod (pH=2.3) and embryos were fixed on coated slides^61^ in 3.7% formaldehyde and stained with PhosphoMyosin-II (Cell Signaling TECHNOLOGY, Ref #3671) diluted 1:200, pericentrin (BD Biosciences, Ref 611814) diluted 1:500 and mounted in Prolongold with DAPI^7^. Images were acquired using a Leica SP5 confocal inverted microscope using a Plan-APO × 63/1.25 NA objective with Z-steps every 1 μm.

### Viscosity measurements

The zona pellucida of embryos was removed using Acid Tyrod (pH=2.3). Cells were aspirated in a 50-μm diameter micropipette until they were completely inside the pipette. Great care was taken that all cells stay inside the pipette for the same duration (∼100 s). They were then released and the shape recovery was recorded. The curve representing the evolution of the reduced diameter 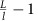 with *L* the bigger diameter and *l* the smaller diameter was plotted as a function of time and fitted as an exponential. The characteristic time *τ* was extracted and the viscosity *η* was deduced from the formula 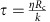 with *R*_c_ as the cell radius and *k* as the cortical tension.

### Statistical analysis

The statistical analysis was performed using GraphPad Prism version 6.00 for MacOS, GraphPad Software, La Jolla, CA, USA (www.graphpad.com). For comparison of several means, the normality of the variables was checked and parametric (Student's *t*-tests with Welch correction where indicated or one-way analysis of variance) or non-parametric comparison tests were performed with a confidence interval of 95%. For contingency analysis, *χ*^2^ or Fischer exact tests were used with a confidence interval of 95%. In all figures, ‘*' corresponds to a *P* value <0.05, ‘**' to a *P* value <0.005, ‘***' to a *P* value <0.0005 and ‘****' to a *P* value <0.0001; NS, not statistically significant. In all figure legends, s.e.m. stands for standard error of the mean and s.d. for standard deviation.

## Additional information

**How to cite this article:** Chaigne, A. *et al.* F-actin mechanics control spindle centring in the mouse zygote. *Nat. Commun.* 7:10253 doi: 10.1038/ncomms10253 (2016).

## Supplementary Material

Supplementary Figures Supplementary Figures 1-5

Supplementary Movie 1 Control embryo expressing His-RFP (purple, Z-projection over 20 μm). The first two frames are 12 hours apart then one picture is shown every 1 hour.

Supplementary Movie 2 Control embryo during pronuclei migration expressing GFP-UtrCH (black). One Z-plane is shown every 564 ms.

Supplementary Movie 3 Control embryo during pronuclei migration. One Z-plane is shown every 300 ms.

Supplementary Movie 4 Control embryo during early pronuclei migration injected with inert fluorescent latex beads (white), 2-3h after pronuclei formation. One Z-plane is shown every 556 ms.

Supplementary Movie 5 Embryo expressing Myosin-Vb tail during pronuclei migration. One Z-plane is shown every 300 ms.

Supplementary Movie 6 Embryo expressing His-RFP (purple, Z-projection over 20 μm) and Myosin-Vb tail. The two pronuclei are initially cortical and close to each other. The first picture is shown 4h after fertilization, then 1 picture is shown every 1h.

Supplementary Movie 7 Embryo expressing His-RFP (purple, Z-projection over 20 μm) and Myosin-Vb tail. The two pronuclei are initially cortical and far from each other. The first picture is shown 4h after fertilization, then 1 picture is shown every 1h.

Supplementary Movie 8 Control embryo during late pronuclei migration injected with inert fluorescent latex beads (white), 10-12h after pronuclei formation. One Z-plane is shown every 556 ms.

Supplementary Movie 9 Control embryo expressing His-RFP (purple, Z-projection over 20 μm) from NEBD until anaphase. One picture is shown every 30 minutes.

Supplementary Movie 10 Control embryo expressing GFP-UtrCH (black). One Z-plane is shown every 20 minutes.

Supplementary Movie 11 Embryo treated with 1 μg/mL Cytochalasin D around NEBD expressing His-RFP (purple, Z-projection over 20 μm) from NEBD. One picture is shown every 30 minutes.

Supplementary Movie 12 Embryo treated with 1 μM Nocodazole around NEBD expressing His-RFP (purple, Z-projection over 20 μm) from NEBD. One picture is shown every 30 minutes.

Supplementary Movie 13 Control embryo before and during the first mitosis. One Z-plane is shown every 1 hour.

Supplementary Movie 14 Control embryo expressing SF9-GFP (Myosin-II intrabody, blue: lower intensity, orange: higher intensity). One Z-plane is shown every 1 hour.

Supplementary Movie 15 Embryo expressing His-RFP (purple, Z-projection over 20 μm) together with cVCA from NEBD until anaphase. One picture is shown every 30 minutes.

Supplementary Movie 16 Embryo treated with 1 μg/mL Cytochalasin D in metaphase expressing His-RFP (purple, Z-projection over 20 μm) from NEBD. One picture is shown every 30 minutes.

Supplementary Movie 17 Embryo treated with 1 μM Nocodazole in metaphase expressing His-RFP (purple, Z-projection over 20 μm) from NEBD. One picture is shown every 30 minutes.

Supplementary Movie 18 Embryo treated with 1 μg/mL Cytochalasin D and 1 μM Nocodazole in metaphase expressing His-RFP (purple, Z-projection over 20 μm) from NEBD. One picture is shown every 15 minutes.

Supplementary Movie 19 Control embryo during mitosis injected with inert fluorescent latex beads (white), more than 13h after pronuclei formation. One Z-plane is shown every 556 ms.

## Figures and Tables

**Table 1:** Cortical tension and viscosity in oocytes and embryos.

	**Early meiosis I oocyte**	**Late meiosis I oocyte**	**Pronucleus stage embryo**	**Mitotic stage embryo**
Cortical tension (nN μm^−1^)	0.92±0.2 (from ref. 12)	0.034±0.1 (from ref. 12)	0.35±0.04	0.61±0.05
Cytoplasmic viscosity (Pa·s)			4.4 × 10^2^±40	12 × 10^2^±300

For cortical tension in embryos, mean of 13 Pronucleus stage and 9 mitotic embryos is shown, measured over 8 independent experiments. The statistical significance of differences is assessed with a *t*-test (*P* value 0.0011). The values of cortical tension for early and late oocytes are extracted from ref. 12. For cytoplasmic viscosity, mean of 16 Pronucleus stage and 15 mitotic embryos is shown, measured over 6 independent experiments. Statistical significance of differences is assessed with a Mann–Whitney test (*P* value <0.0001).
