# Supplementary material for: F-actin mechanics control spindle centring in the mouse zygote
Source: Nat Commun. 2016 Jan 4;7:10253. doi: 10.1038/ncomms10253 (PMC4725770; doi:10.1038/ncomms10253)
Supplement: Supplementary Figures — 1-5 [file ncomms10253-s1.pdf]

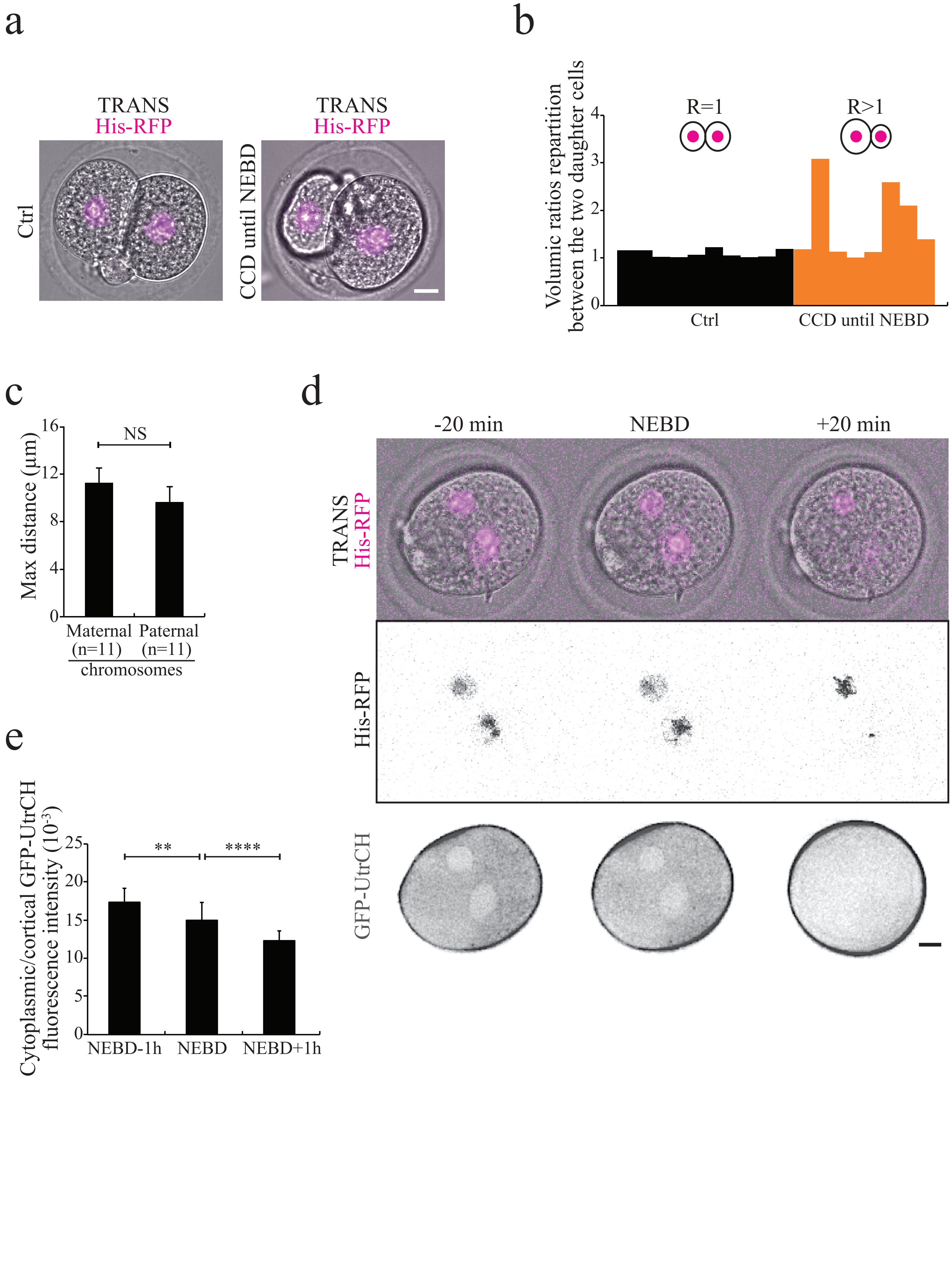

Supp Figure 1

Supplementary Figure 1:

a) Embryos treated (right panel) or not (left panel) with 1 µg/mL Cytochalasin D (CCD) during pronuclei migration until NEBD expressing His-RFP (purple, Z-projection over 100 µm) at the 2-cell stage. Scale bar: 10 µm. b) Bar graph showing the repartition of the ratios between the volumes of the two daughter cells for controls (black bars) or embryos treated with CCD during pronuclei migration until NEBD (orange bars). c) Graph showing the maximum distance (i.e the distance between the points furthest apart on the trajectories) of chromosome motion in control embryos during the migration of the two sets of chromosomes towards the embryo center (female: left bar; male: right bar). Mean of 11 embryos are shown over 7 independent experiments. SEM is plotted on each bar. Statistical significance of differences is assessed with a Mann-Whitney test (P-value 0.327). d) Control embryo expressing His-RFP (upper and middle panels, purple or black, Z-projection over 20 µm) and GFP-UtrCH (lower panel, black, one Z-plane is shown). One picture is shown every 20 minutes. Scale bar: 10 µm. e) Bar graph showing the relative cytoplasmic fluorescence intensity in embryos expressing GFP-UtrCH at NEBD-1h, NEBD, NEBD+1h. Mean of 19 embryos is shown over 3 independent experiments. SD is plotted on each bar. Statistical significance of differences is assessed with a t-test or a t-test with Welch correction (P-values: NEBD-1h/NEBD: 0.0019; NEBD/NEBD+1h <0.0001).

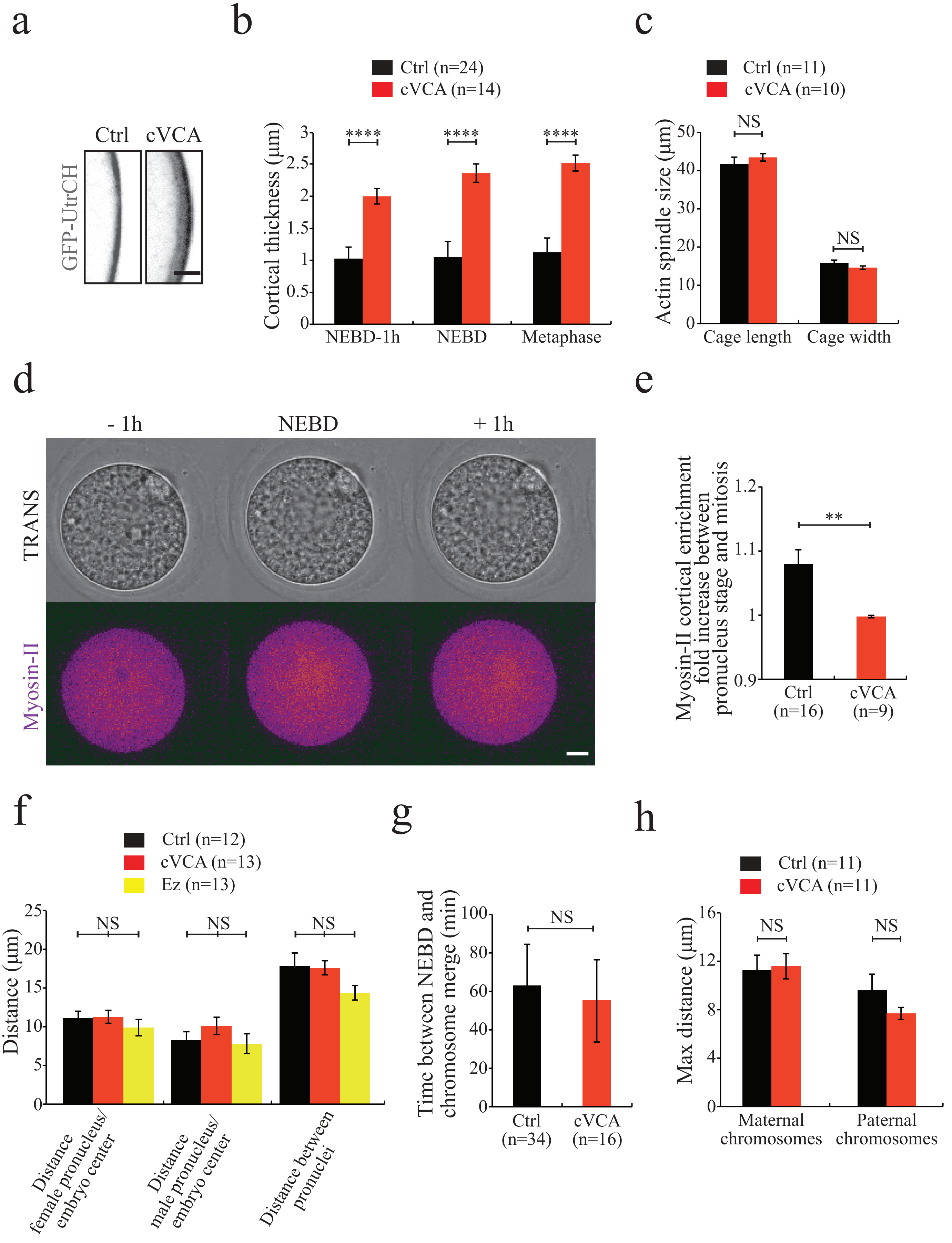

Supp Figure 2

Supplementary Figure 2:

a) Cortex of embryos expressing GFP-UtrCH (black) alone or with cVCA (right panel). One Z-plane is shown. Scale bar: 3 μm. b) Graph bar showing the cortical thickness of embryos expressing cVCA or not. Mean is shown for 24 controls and 14 cVCA embryos from 7 independent experiments. Statistical significance of differences is assessed with t-tests with Welch correction (All P-values <0.0001). c) Graph bar showing measurements of the F-actin cage width and length of embryos expressing cVCA or not. Mean is shown for 11 controls and 10 cVCA embryos from 8 independent experiments. Statistical significance of differences is assessed with a t-test or a Mann-Whitney test (P-values: length 0.4177; width 0.0685). d) Embryo expressing cVCA and SF9-GFP (blue: lower intensity; orange: higher intensity, one Z-plane). Scale bar: 10 μm. e) Graph bar showing Myosin-II cortical recruitment increase between Pronucleus stage and mitosis for controls and cVCA embryos. Mean is shown for 16 controls and 9 cVCA embryos from 2 independent experiments. Statistical significance of differences is assessed with a t-test with Welch correction (P-value 0.0025). f) Bar graph showing pronuclei distance to the embryo center and to each other 1 hour before NEBD for controls, cVCA or Ezrin expressing embryos. Mean is shown of 12 controls, 13 cVCA and 13 Ezrin embryos from 6 independent experiments. Statistical significance of differences is assessed with Anovas (P-values: female pronuclei/embryo center: 0.5115; male pronuclei/embryo center: 0.3273; between pronuclei: 0.075). g) Bar graph showing the time from NEBD to chromosome merge. Mean is shown of 34 controls and 16 cVCA embryos from 6 independent experiments. SD is plotted on each bar. Statistical significance of differences is assessed with a Mann-Whitney test (P-value 0.1875). h) Graph showing the maximum distance of chromosome motion in controls (from Supplementary Figure 1c) and cVCA embryos. Mean of 11 controls and 11 cVCA embryos are shown over 4 independent experiments. Statistical significance of differences is assessed with a t-test or a Mann-Whitney test (P-values: female 0.8433; male 0.5104). For all graphs except g) SEM is plotted on each bar. Controls: black bars, cVCA: red bars, Ezrin: yellow bars.

a

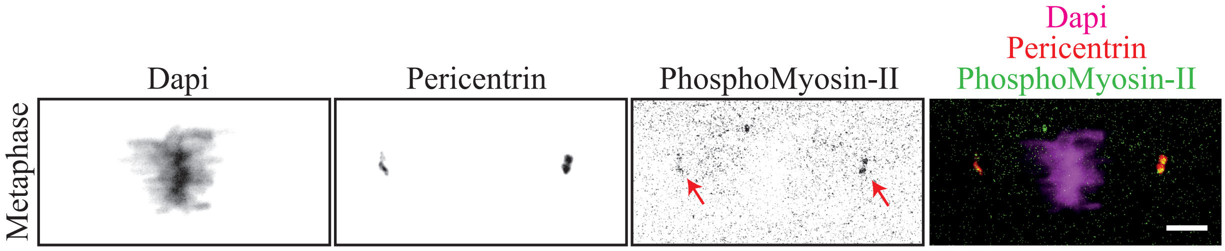

b

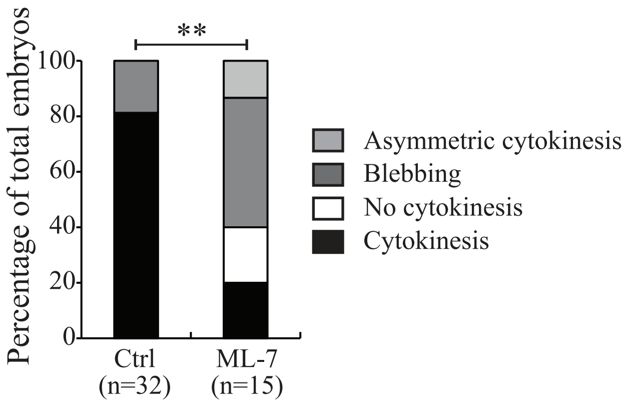

c

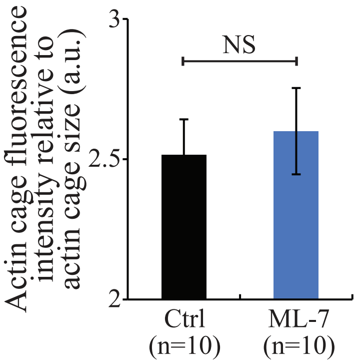

Supp Figure 3

Supplementary Figure 3:

a) Control embryo during mitosis stained for chromosomes (Dapi, black; merge, magenta; a Z-projection is shown over 9 μm), pericentrin (Pericentrin, black; merge, red; a Z-projection is shown over 3 μm) and PhosphoMyosin-II (PhosphoMyosin-II, black; merge, green; a Z-projection is shown over 3 μm). Red arrows point to the PhosphoMyosin-II foci. Scale bar: 5 μm. b) Bar graph showing the percentage of embryos achieving normal cytokinesis, asymmetric cytokinesis, blebbing or no cytokinesis, for 32 controls and 15 embryos treated with 30 μM ML-7 in metaphase from 2 independent experiments. The statistical significance of differences is assessed with a Fisher test (P-value 0.0077). c) Bar graph showing the fluorescence of the F-actin cage in embryos expressing GFP-UtrCH relative to the size of the cage for control embryos and embryos treated with 30 μM ML-7 in metaphase. Mean is shown of 10 controls and 10 embryos treated with ML-7 over 2 independent experiments. SEM is plotted one each bar. Statistical significance of differences is assessed with a t-test (P-value 0.6746).

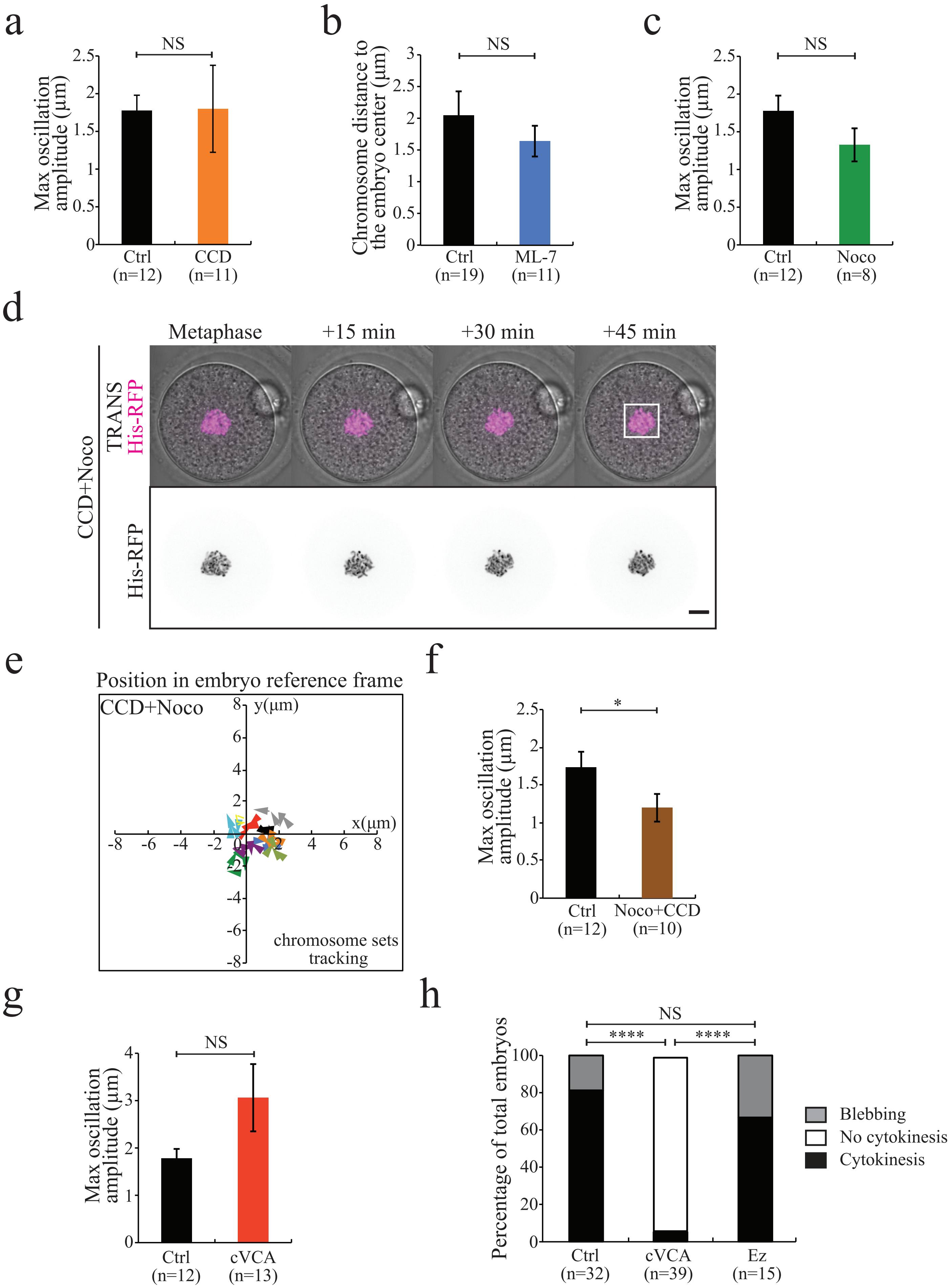

Supp Figure 4

Supplementary Figure 4:

a) Graph showing the maximum amplitude of chromosome motion in embryos treated or not with with Cytochalasin D (CCD) in metaphase. Mean of 12 controls and 11 CCD embryos are shown. Statistical significance of differences is assessed with a t-test with Welch correction (P-value 0.974). b) Graph showing the chromosome distance to the embryo center in metaphase for embryos treated with ML-7 or not. Mean is shown of 19 controls and 11 ML-7 embryos from 2 independent experiments. Statistical significance of differences is assessed with a Mann-Whitney test (P-value 0.9142). c) Graph showing the maximum amplitude of chromosome motion in embryos treated or not with Nocodazole in metaphase. Mean of 12 controls and 8 Nocodazole embryos are shown over 2 independent experiments. The statistical significance of differences is assessed with a t-test with Welch correction (P-value 0.1538). d) Embryo treated with CCD+Nocodazole in metaphase expressing His-RFP (purple, black, Z-projection over 20 μm). Scale bar: 10 μm. e) Graph showing the position of the centroid of the chromosomes from metaphase for embryos treated with CCD+Nocodazole in metaphase. Each color represents an embryo, arrows point to the last position recorded, one time point every 15 minutes for 1 hour. f) Graph showing the maximum amplitude of chromosome motion in embryos treated with CCD+Nocodazole in metaphase or not. Mean of 12 controls and 10 CCD+Nocodazole embryos are shown over 2 independent experiments. The statistical significance of differences is assessed with a Mann-Withney test (P-value 0.03). g) Graph showing the maximum amplitude of chromosome motion in embryos expressing cVCA or not. Mean of 12 controls and 13 cVCA embryos are shown over 4 independent experiments. Statistical significance of differences is assessed with a t- test with Welch correction (P-value 0.1046). h) Graph showing the percentage of embryos achieving normal cytokinesis, blebbing or no cytokinesis, for 32 controls, 39 cVCA and 15 Ezrin expressing embryos from 3 independent experiments. Statistical significance of differences is assessed with a Fisher test or a χ^2 test (P-values: control/ cVCA <0.0001; control/ Ez 0.7428; cVCA/ Ez <0.0001). For all graphs except h) SEM is plotted on each bar.

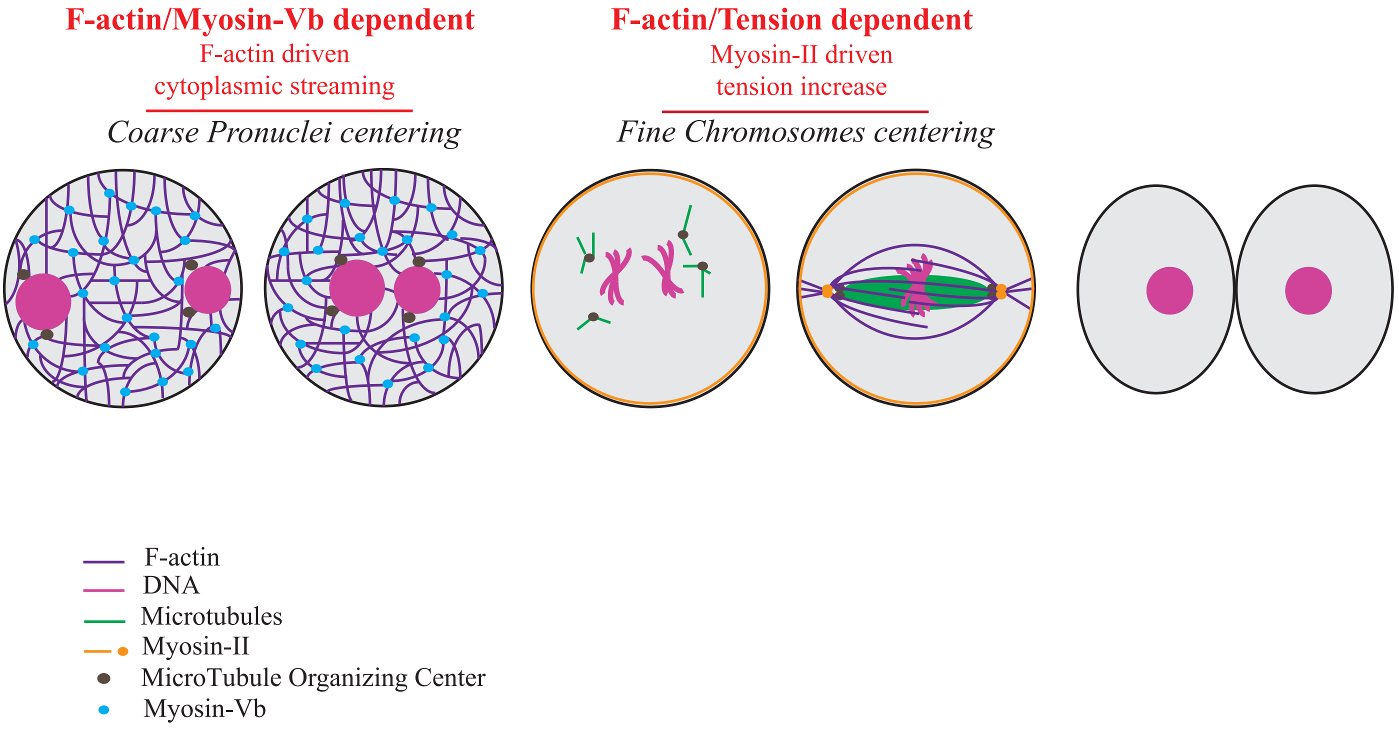

Supp Figure 5

Supplementary Figure 5: Model for acentrosomal mitotic spindle positioning in mouse embryo. Violet: F-actin, magenta: DNA, green: microtubules, brown: microtubules organizing centers, orange: Myosin-II, blue: Myosin-Vb.
